# The Ras Antagonist, Farnesylthiosalicylic Acid (FTS), Decreases Fibrosis and Improves Muscle Strength in *dy^2J^/dy^2J^* Mouse Model of Muscular Dystrophy

**DOI:** 10.1371/journal.pone.0018049

**Published:** 2011-03-22

**Authors:** Yoram Nevo, Shlomit Aga-Mizrachi, Edva Elmakayes, Nurit Yanay, Keren Ettinger, Moran Elbaz, Zivia Brunschwig, Oshrat Dadush, Galit Elad-Sfadia, Roni Haklai, Yoel Kloog, Joab Chapman, Shimon Reif

**Affiliations:** 1 Pediatric Neuromuscular Laboratory and the Neuropediatric Unit, Hadassah Hebrew University Hospital, Jerusalem, Israel; 2 Department of Neurobiochemistry, The George S. Wise Faculty of Life Sciences, Tel-Aviv University, Tel-Aviv, Israel; 3 Department of Neurology, Sheba Medical Center, Tel-Aviv University, Tel-Aviv, Israel; 4 Pediatric Department, Dana Children's Hospital, Tel-Aviv Sourasky Medical Center, Tel-Aviv University, Tel-Aviv, Israel; University of Pennsylvannia, United States of America

## Abstract

The Ras superfamily of guanosine-triphosphate (GTP)-binding proteins regulates a diverse spectrum of intracellular processes involved in inflammation and fibrosis. Farnesythiosalicylic acid (FTS) is a unique and potent Ras inhibitor which decreased inflammation and fibrosis in experimentally induced liver cirrhosis and ameliorated inflammatory processes in systemic lupus erythematosus, neuritis and nephritis animal models. FTS effect on Ras expression and activity, muscle strength and fibrosis was evaluated in the *dy^2J^*/*dy^2J^* mouse model of merosin deficient congenital muscular dystrophy. The *dy^2J^*/*dy^2J^* mice had significantly increased RAS expression and activity compared with the wild type mice. FTS treatment significantly decreased RAS expression and activity. In addition, phosphorylation of ERK, a Ras downstream protein, was significantly decreased following FTS treatment in the *dy^2J^*/*dy^2J^* mice. Clinically, FTS treated mice showed significant improvement in hind limb muscle strength measured by electronic grip strength meter. Significant reduction of fibrosis was demonstrated in the treated group by quantitative Sirius Red staining and lower muscle collagen content. FTS effect was associated with significantly inhibition of both MMP-2 and MMP-9 activities. We conclude that active RAS inhibition by FTS was associated with attenuated fibrosis and improved muscle strength in the *dy^2J^*/*dy^2J^* mouse model of congenital muscular dystrophy.

## Introduction

Merosin deficient congenital muscular dystrophy (MDC1A, OMIM # 607855) is the most common form of the congenital muscular dystrophies. It is an autosomal recessive disorder caused by mutations in the LAMA2 gene, localized to chromosome 6q22–q23. Most children affected with this disorder have severe clinical symptoms. They do not achieve independent ambulation and die in childhood or early adulthood [1], [2]. The Lama2^dy-2J^ (*dy^2J^/dy^2J^*) is a useful mouse model for MDC1A. It has a spontaneous G to A mutation in the donor splice site of exon 2 which results in exon skipping [3], [4], [5]. The clinical course of the homozygous *dy^2J^/dy^2J^* mouse involves early onset progressive muscle weakness and motor deterioration; though less severe than its allelic form the *dy/dy* mouse. Muscle biopsy shows progressive dystrophic changes including muscle fiber necrosis, regeneration, and progressive fibrosis [4], [6]. Comparable to merosin deficient congenital muscular dystrophy children, *dy^2J^/dy^2J^* mice demonstrate a peripheral neuropathy in addition to the muscular dystrophy [7], [8], [9].

The Ras superfamily of guanosine-triphosphate (GTP) binding proteins that includes more than 50 members regulates a diverse spectrum of intracellular processes [10]. Ras proteins are expressed in almost all adult and fetal tissues, acting as molecular switches, and activating signal transduction pathways that regulate cellular proliferation, differentiation and survival [11]. They are attached to the inner side of the plasma membrane where they are activated by cell surface receptors to induce the conversion of the inactive Ras, guanosine-diphosphate (GDP), to active Ras-GTP [12]. Over expression of Ras proteins causes proliferation and tumor genesis. In addition, previous studies demonstrated increased Ras expression in inflammatory processes, such as systemic lupus erythematosus (SLE), neuritis and nephritis [13], [14], [15]. Ras has also been shown to be involved in the modulation of the immune response. It affects the expression of major histocompatibility complex (MHC) molecules, antigen processing, cytokine production, and regulation of receptors, T cells, and growth factors [16].

Farnesythiosalicylic acid (FTS) is a synthetic derivative of carboxylic acid, which structurally resembles the carboxy-terminal farneslcysteine group common to all Ras proteins. It acts as a functional Ras antagonist, affecting Ras membrane interactions by dislodging the protein from its anchorage domains, facilitating its degradation, and thus reducing the cellular Ras content and the cells' response to it [17], [18]. FTS is a potent growth inhibitor of cells expressing active H-Ras, K-Ras, or N-Ras *in vitro* and of human pancreatic and colon carcinoma as well as hematologic malignancies and melanoma tumors [19], [20]. We have previously shown that FTS is a reversible drug [19], [20], [21] with reversibility of its inhibitory effects on Ras-dependent growth *in vitro* [18], [19], [20] and *in vivo* [21] and on Ras -dependent behavior *in vivo* [15]. Moreover, FTS has been found to be beneficial in decreasing inflammation and fibrosis in experimentally induced liver cirrhosis and in treating and preventing inflammatory processes such as SLE, neuritis or nephritis in animal models [13], [14], [15], [22], [23].

In the current study, given previous documentation of anti-inflammatory and anti-fibrotic characteristics of FTS, the role of Ras and the therapeutic potential of FTS was evaluated in this mouse model of merosin deficient congenital muscular dystrophy. FTS treatment significantly decreased muscle Ras expression and activity and was associated with significant reduction of fibrosis and improvement in hind limb muscle strength in the *dy^2J^/dy^2J^* mice.

## Materials and Methods

### Mice

C57BL/6J Lama2^dy-2J^ (*dy^2J^/dy^2J^*) heterozygote mice were obtained from Jackson Laboratories (Bar Harbor, Maine, USA) and were bred at the Hebrew University SPF animal housing facility. This study was carried out in strict accordance with the recommendations in the Guide for the Care and Use of Laboratory Animals of the National Institutes of Health. The protocol was approved by the Committee on the Ethics of Animal Experiments of the Hebrew University (Permit Number: 122.03–04). All surgery was performed under ketamine-xylazine anesthesia, and all efforts were made to minimize suffering. Mice were maintained under standard conditions, 23±1°C, 12 h light cycle (7 a.m.–7 p.m.) with ad libitum access to food and drink. No animal had paralysis with inability to reach food or water or dramatic weight loss (more than 10% weight loss between two weighings, or more than 20% from initial weight) or any other severe stress signs requiring withdrawal from the study. No other side effects were noted in the treatment group. Delineation between the *dy^2J^/dy^2J^* affected mice, heterozygous for the lama2 gene mutation and wild type C57BL/6J (WT) mice was detected by PCR reaction with the following primers: forward 5′-TCCTGCTGTCCTGAATCTTG and reverse 5′- CTCTATTACTGAACTTTGGATG. The digestion of the PCR products with the NdeI restriction enzyme (recognition sequence: CATATG) resulted in characteristic product size for each of the mice genotypes [4].

Following previous protocols in the rat model of liver cirrhosis [22], WT and *dy^2J^/dy^2J^* mice were injected intra-peritoneally 3 times a week with FTS 5 mg/kg or control solution (see below), for 12 weeks from the age of 6 weeks (n = 7/group, each group consisted of 4 males and 3 female mice). At the end of the study both hind limb muscles were dissected. Part of the muscle sample was frozen in liquid nitrogen and stored at −80°C for biochemical analysis. Quadriceps femoris muscle was rapidly frozen in isopentane pre-chilled by liquid nitrogen for cryostat sections and histology.

### Preparation of Farnesylthiosalicylic Acid (FTS)

FTS was a gift from Concordia Pharmaceuticals (http://www.concordiapharma.com). FTS was prepared as previously described [24]. For each set of experiments, FTS was prepared as a 0.1 M stock solution in chloroform, the chloroform was removed from the stock by a nitrogen stream prior to use, and the dry FTS then dissolved in ethanol. The FTS/ethanol solution was alkalinized by the addition of 1N NaOH and then diluted by the addition of phosphate-buffered saline (PBS). The control solution was prepared as described above except that FTS and NaOH were excluded.

### Muscle strength

Total peak force (in gram force/gram bodyweight) was determined once a week using an electronic Grip Strength Meter, Columbus Instruments (Columbus, OH, USA). Each week muscle strength measurements of both fore and hind limbs were performed according to Dadush O et al. [25], with five measurements done on each fore and hind limb from each animal. The three highest measurements were averaged to give the strength score. The mice were allowed to rest for 10 minutes between fore and hind limb measurements. All measurements were performed by the same examiner.

### Mobility

At the end of the study the mice were video recorded for 10 minute sessions which were analyzed by the Ethovision XT Behavioral activity system (version 5, Noldus Information Technology, Wageningen, the Netherlands) [25]. The test arena was transparent, 27 cm wide, 48 cm long and 25 cm high. To reduce body movements which were not associated with mobility, only recordings which resulted in movement of at least 0.5 cm in 0.2 sec were included in the calculated data. Total distance and mean velocity as well as maximal distance and velocity in 0.2 seconds were calculated using the software.

### Histology and quantitative analysis of fibrosis

Quadriceps muscle biopsies were flash-frozen in cooled isopentane and mounted in “Tissue-Teck”. Ten µm tissue sections were stained with Hematoxylin-Eosin (H&E) for histology, and with Sirius red counterstained with fast green for collagen quantitative measurement using Ariol SL-50 automated image analysis (Applied Imaging, CA, USA). The Ariol software was set-up to distinguish between the red (R), fibrotic (collagen stained) area, and green (G), non-collagen area. The fibrosis percentage was calculated by dividing the fibrotic area (R) by the total area (R+G). The mean of the fibrotic area was calculated from five regions in each biopsy.

### Collagen measurement

Total muscle collagen content was quantitatively measured by the Sircol™ Collagen Assay (Biocolor, Newtownabbey, Northern Ireland). This is a quantitative dye-binding method analyzing acid-soluble collagens (Type I–IV), extracted from mammalian tissues. Soluble collagen quantification was performed from 30 mg of hind limb muscle according to manufacturer's manual.

### Western Blotting and Ras activity

**Figure 1:**
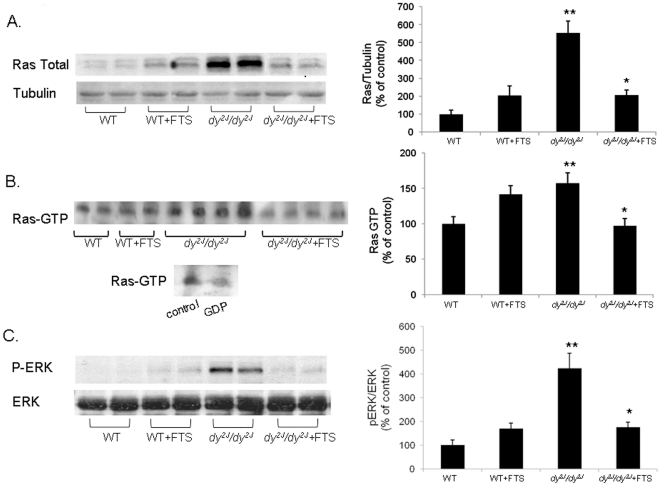
The effect of FTS on Ras expression, activity and pERK. Western blot analysis gels and densitometry graphs of Ras expression (A) Ras activity (B) and p-ERK (C) in the WT and *dy^2J^/dy^2J^* mice are presented. Ras expression, activity and pERK measurements were significantly lower in the FTS treated compared to the untreated *dy^2J^/dy^2J^* mice (* A; *P*<0.01 B; *P*<0.05 C; *P*<0.02 Student's *t*-test). All 3 measurements were higher in the *dy^2J^/dy^2J^* compared to the WT mice (** A; *P*<0.01 B; *P*<0.01 C; *P*<0.02; Student's *t*-test). The lower panel of figure B is the negative control for Ras-GTP binding assay. Each bar represents the mean ± SEM of 4 independent experiments from six mice.

In order to examine the effect of FTS on Ras cascade in muscle, total Ras expression and ERK phosphorylation were determined in a 30 µg samples by immunoblotting with pan anti-Ras (Calbiochem, La Jolla, CA), pERK (Sigma, St. Louis, MO, USA), ERK (Santa Cruz, CA, USA)and goat anti-mouse HRP (Bio-Rad, Richmond, CA, USA) antibodies followed by enhanced chemiluminescence (ECL) [26]. Normalization was performed by anti β-tubulin (Sigma). Since Ras-GTP activates Raf in the signal transduction pathway, Ras activity was determined by pull-down of Ras-GTP by Ras binding domain (RBD) of Raf, which are conjugate to GST-gluttion-agarose beads. Briefly, Lysates containing 800 µg protein were used to determine the Ras-GTP by using glutathione S-transferase (GST) Ras-binding domain pull-down assay [26]. Following 30 min rotation at 4°C the sample were washed 3 times with buffer. The proteins were dissolved in SDS sample buffer and separated by SDS page which was followed by immunoblotting with pan anti-Ras antibody and goat anti-mouse HRP followed by ECL [26]. A negative control for the assay was incubating the Ras in homogenates of WT mice with 1 mM GDP for 30 min to induce GDP for GTP exchange. We then subjected the lysates to the pool down assay. This treatment caused exchange of most of the GTP to GDP on Ras and a 90% reduction in Ras-GTP binding was observed (Figure 1B lower panel).

### Gelatin Zymography for MMP-2 and MMP-9

MMP-2 and MMP-9 activity was determined from mice muscles as previously described [27], with minor modifications. Briefly, samples of 50 µg homogenated muscle were separated on gelatin-impregnated (1 mg/ml, Difco, Detroit, MI), SDS-8% polyacrylamide gels under non-reducing conditions, followed by 30 min shaking in 2.5% Triton X-100 (BDH, UK). The gels were then incubated for 16 h at 37°C in 50 mM Tris, 0.2 M NaCl, 5 mM CaCl_2_, 0.02% Brij 35(w/v) at pH 7.6. At the end of incubation the gels were stained with 0.5% Coomassie G 250 (Bio-Rad) in methanol/acetic acid/H_2_O (30∶10∶60). The intensity of the various bands was determined on a computerized densitometer (Molecular Dynamics, type 300A).

### Statistical analysis

All data are expressed as mean ± standard error of the mean (SEM) and all statistical analysis was completed in SPSS (SPSS 15.0 for windows). Statistical analysis for direct comparison between two groups was performed by unpaired Student's *t*-test and non parametric Mann-Whitney test. Multiple comparisons between groups were made using repeated-measures ANOVA. Significance was set at *P*<0.05 for all comparisons.

## Results

### Ras expression and Ras-GTP

WT and *dy^2J^/dy^2J^* mice were treated with either FTS or control for 12 weeks. Immunoblotting of skeletal muscle was carried out at the end of the study using anti-Ras antibody. Untreated *dy^2J^/dy^2J^* mice showed significantly higher Ras expression compared to the WT group (555.08±66.32 vs. 100±22.45 densitometry, percent of control; *P*<0.01; Figure 1A). FTS treatment was associated with significantly decreased Ras expression in the *dy^2J^/dy^2J^* mice (205.76±19.37; *P*<0.01). Treatment of WT mice with FTS caused an increase in Ras expression which was by far much lower than the increase observed in the *dy^2J^/dy^2J^* mice (206.76±29.37). In addition to Ras expression, Ras activity was measured at the end of the study by the Ras binding domain pull-down assay. In this assay the GTP-bound Ras is detected by its preferential binding to the RBD domain of Raf1 which is conjugated to sepharose beads [26]. We found that Ras-GTP levels were higher in *dy^2J^/dy^2J^* compared to the levels in WT mice (157.40±14.53 vs. 100±10.23; *P*<0.05. Figure 1B). The levels of Ras-GTP were significantly reduced in the FTS treated *dy^2J^/dy^2J^* group (97.16±10.54; *P*<0.01). Moreover, Ras-GTP in the treated *dy^2J^/dy^2J^* normalized and was comparable to the WT group. Treatment of the WT mice with FTS induced an increase in Ras-GTP (141.72±12.4) comparable to the increase in Ras expression in these mice. The nature of these increases observed in the WT mice is not known.

In addition, we measured the phosphorylation of ERK, a Ras downstream protein (Figure 1C). ERK phosphorylation was very high in the *dy^2J^/dy^2J^* mice compared to the WT group (424.97±63.85 vs. 100±21.56; *P*<0.02) while, FTS treatment significantly decreased this phosphorylation (175.62±21.53; *P*<0.02). Small increase in pERK was noted in the WT FTS (169.29±31.1) while, total ERK did not change at all.

### Muscle strength

**Figure 2:**
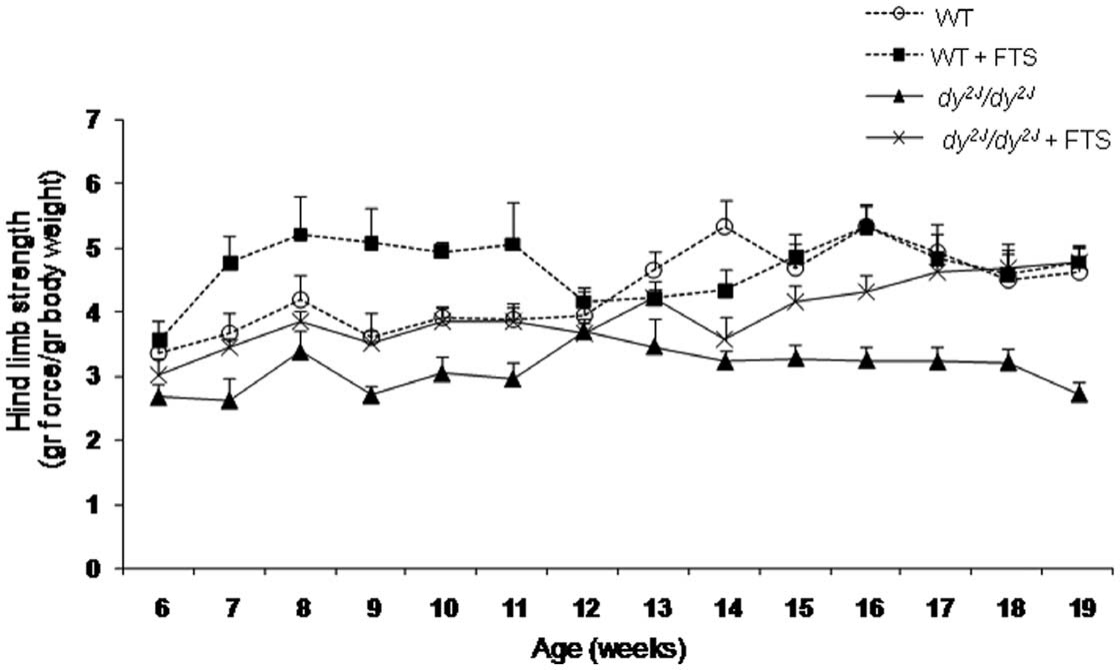
Hind limb muscle strength in WT and *dy^2J^/dy^2J^* FTS treated and untreated groups. The data of seven mice in each group is expressed as mean ± SEM. Repeated measures ANOVA test showed significant improved hind limb strength in the treated *dy^2J^/dy^2J^* mice (*P*<0.05). There was no significant difference between the treated and untreated WT groups.

Total peak force was determined once a week using an electronic Grip Strength Meter. Significant difference in hind limb muscle strength was detected between the untreated *dy^2J^/dy^2J^* and the WT mice throughout the study (*P*<0.01; Figure 2). A significant increase in hind limb muscle strength was noted in the FTS treated compared to the untreated *dy^2J^/dy^2J^* mice (*P*<0.05). During the study period muscle strength increased from 3.01±0.27 to 4.79±0.22 (gram force/gram body weight) in the treated *dy^2J^/dy^2J^* mice, while remained unchanged from 2.67±0.22 to 2.72±0.19 in the untreated *dy^2J^/dy^2J^* group. Moreover, at the end of the trial the hind limb muscle strength of the *dy^2J^/dy^2J^* treated mice completely normalized to that of both WT groups (4.79±0.22 vs. WT: 4.64±0.39; WT+FTS: 4.77±0.25). Such a difference was not detected in the fore (stronger) limbs of the *dy^2J^/dy^2J^* mice (Figure S1), and in the fore and hind limbs of the treated and untreated WT groups.

### Muscle histology

**Figure 3:**
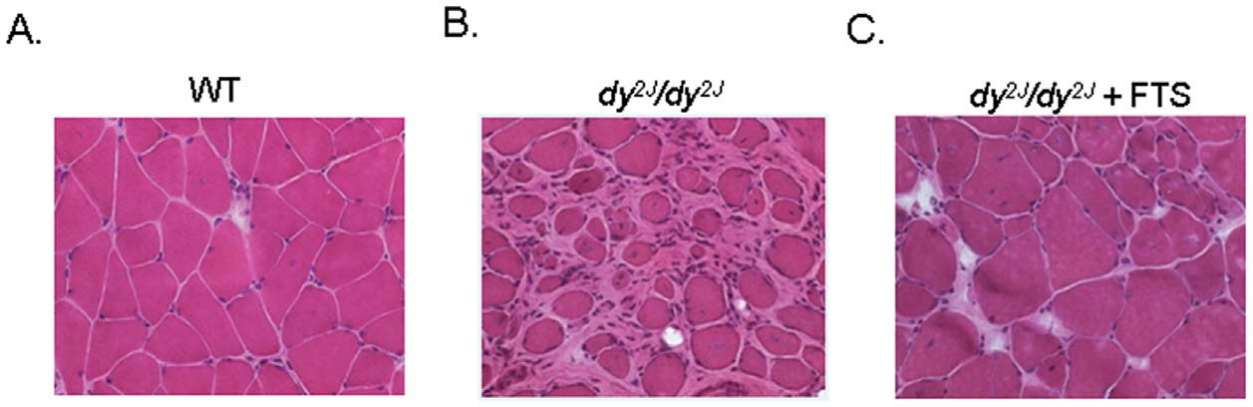
The effect of FTS on muscle pathology. Digital images of hematoxylin and eosin stained muscle. (A) WT untreated mouse presenting normal organization of muscle tissue with no abundant connective tissue. (B) Untreated *dy^2J^/dy^2J^* mouse showing severe dystrophic changes with abnormal variation of fiber size, internal nuclei and severe fibrosis. (C) Treated *dy^2J^/dy^2J^* mouse, showing still variation in fiber size. Magnification ×400.

In comparison with the normal WT (Fig. 3A), Hematoxylin and eosin staining of untreated *dy^2J^/dy^2J^* mouse quadriceps muscle at the age of 18 weeks showed severe advanced dystrophic changes with abnormal variation of fiber size, internal nuclei and severe excessive fibrosis (Fig. 3B). The FTS treated *dy^2J^/dy^2J^* muscle showed considerable fibrosis attenuation (see next paragraph) but still abnormal myopathic changes with variation in fiber size, and increased number of central nuclei (Fig. 3C).

### Quantitative muscle fibrosis by Sirius red staining

**Figure 4:**
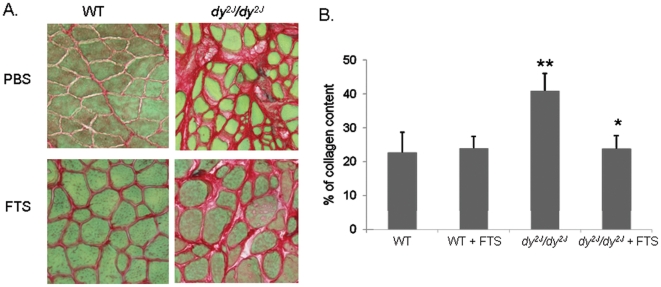
The effect of FTS on muscle histological fibrosis. (A) Digital images of sirius red stained muscle and (B) quantified collagen content graph. A significant decrease in collagen content was observed in the FTS treated *dy^2J^/dy^2J^* compared to the untreated *dy^2J^/dy^2J^* mice (* *P*<0.02; Student's *t*-test). There was a significant difference between the WT and *dy^2J^/dy^2J^* groups (** P<0.05; Student's *t*-test). Each bar represents the mean ± SEM of five regions in each biopsy of five mice. Magnification ×400.

On Sirius red staining, quantified by the Ariol SL-50 automated image analysis (Fig. 4A and B), untreated *dy^2J^/dy^2J^* mice exhibited significantly higher total quadriceps collagen content compared to the WT mice (40.92±5.16 vs. 22.76±5.89; *P*<0.05). A significant decrease in collagen was noted in the FTS treated *dy^2J^/dy^2J^* compared to the untreated *dy^2J^/dy^2J^* mice (23.87±3.91 vs. 40.92±5.16; *P*<0.02). Collagen accumulation in the FTS treated *dy^2J^/dy^2J^* mice was reduced to the level of both WT groups (23.87±3.91 vs. WT: 22.76±5.89; WT+FTS: 23.93±3.47). No effect of FTS treatment on collagen content was observed in the WT mice.

### Quantitative muscle collagen content by Sircol Collagen Assay

**Figure 5:**
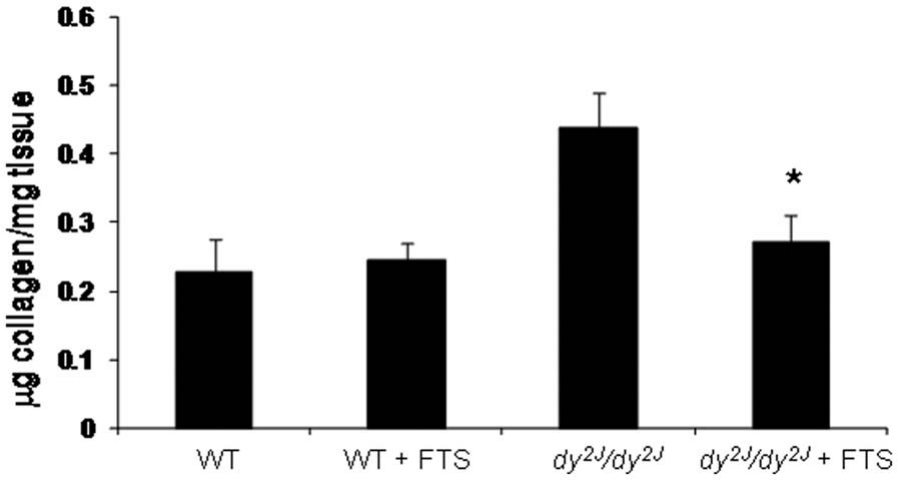
The effect of FTS on muscle collagen content. Sircol collagen assay of muscle biopsies. Student's t-test and non parametric Mann-Whitney test showed a significant decreased in collagen level in the FTS treated *dy^2J^/dy^2J^* compared to the untreated group (*P<0.02; Student's *t*-test). There was a significant difference between the WT and *dy^2J^/dy^2J^* groups (** P<0.01; Student's *t*-test). Each bar represents the mean ± SEM of seven mice.

Quantitative total muscle collagen content using the Sircol Collagen Assay was determined at the end of the study. Significantly higher collagen level was shown in the untreated *dy^2J^/dy^2J^* compared to the WT mice (0.44±0.05 vs. 0.23±0.05 µg collagen/mg tissue; *P*<0.01; Fig. 5). The *dy^2J^/dy^2J^* FTS treated mice showed significantly decreased collagen levels (0.27±0.04, *P*<0.02) compare to the untreated group and were normalized to the treated WT group (0.25±0.02). Collagen levels were similar in the treated and untreated WT groups (0.25±0.02 vs.0.23±0.05).

### MMP-2 and MMP-9 activity

**Figure 6:**
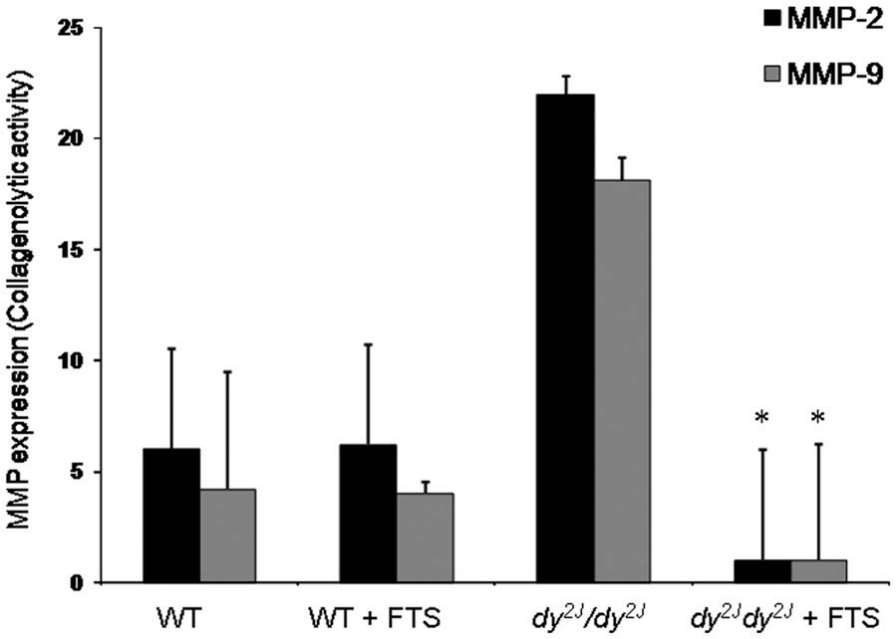
The effect of FTS on MMP-2 and MMP-9 activity. Densitometry graphs of Gelatin Zymography for MMP-2 and MMP-9. The collagenolytic activities of MMP-2 and MMP-9 were significantly lower in the FTS treated *dy^2J^/dy^2J^*compared to the untreated group (*P<0.01; Student's *t*-test). There was a significant difference between the WT and *dy^2J^/dy^2J^* groups (** P<0.05; Student's *t*-test). Each bar represents the mean ± SEM of six mice.

The collagenolytic activities of MMP-2 and MMP-9 were significantly higher in the untreated *dy^2J^/dy^2J^*compared to the WT group (22.2±2.1 vs. 6.1±4.7 and 18.5±2.3 vs. 4.2±4.1 collagenolytic activity, respectively; *P*<0.01; Figure 6). FTS treatment was associated with significantly inhibition of both MMP-2 and MMP-9 activities in the *dy^2J^/dy^2J^* mice (1.5±4.5; 1.4±4.7 respectively, *P*<0.01). Following treatment there was no longer significant difference in MMP-2 and MMP-9 activity between the treated *dy^2J^/dy^2J^* and WT groups.

## Discussion

In muscular dystrophy following recurrent cycles of muscle injury and repair regeneration fails and the contractile tissue is replaced with fibrotic tissue and fat. In advanced stages of the disease the abundant connective tissue (fibrosis) distorts normal muscle architecture and is associated with severe muscle weakness in both human muscular dystrophy patients and *dy^2J^/dy^2J^* mouse model of congenital muscular dystrophy [8], [28]. Fibrous tissue accumulation is characterized by proliferation of fibroblasts, secretion of cytokines and growth factors, increased collagen and other extra cellular matrix proteins. Protooncogens, in particular Ras, play an important role in fibroblast proliferation. It has been proposed that Ras proteins function as intermediates in signal transduction initiated by a large variety of growth factors such as epidermal growth factor (EGF) and platelet – derived growth factor (PDGF) [29], [30]. Ras proteins are attached to the inner side of the plasma membrane where they are activated by cell surface receptors to induce the conversion of the inactive Ras, guanosine-diphosphate (GDP), to active Ras-GTP [12], [31]. S- trans, trans farnesythiosalicylic acid (FTS) is a unique and potent competitive Ras inhibitor. FTS's action is based on its ability to interact with Ras anchorage domains, dislodging the protein from the membrane attachment, and markedly accelerating Ras degradation [32], [33].

The *dy^2J^/dy^2J^* mouse model of merosin deficient congenital muscular dystrophy (MDC1A) is a useful model to study pharmacological intervention on mouse strength and mobility and muscle fibrosis. At the age of 6 weeks *dy^2J^/dy^2J^* mice are significantly weaker than the C57BL/6J WT mice. In the ensuing 12 weeks muscle strength further decreases in the *dy^2J^/dy^2J^* while increasing in the WT [3], [34]. At that age muscle fibrosis significantly increases in the *dy^2J^/dy^2J^* compared to the WT [25].

In this study we showed that FTS treatment significantly decreased both Ras expression and activity in the *dy^2J^/dy^2J^* mouse model of merosin deficient congenital muscular dystrophy. We further showed that phosphorylation of ERK, a Ras downstream protein, was high in *dy^2J^/dy^2J^* mice and was significantly decreased following FTS treatment. Decreased Ras activity and expression was associated with significantly reduced muscle fibrosis studied by quantitative automated image analysis of the biopsies as well as reduced quantitative muscle collagen content. We know from earlier studies that Ras proteins are present in fibroblasts, inflammatory and muscle cells [15], [17]–[19]. Therefore each of these cell populations can potentially be involved in the effects of FTS. Our results are consistent with previous studies which demonstrated effective anti-fibrotic effect of FTS in thioacetamide (TAA) experimentally induced liver cirrhosis in rats [22]. FTS had both preventive qualities by inhibiting hepatic stellate cell (HSC) activation and proliferation, as well as partial reversal effect on liver cirrhosis.

In addition, FTS treated *dy^2J^/dy^2J^* mice had significant improvement in hind limb muscle strength which normalized and was comparable to the WT strength at the end of the study. However, the treated mice had no significant improvement in mouse mobility on motion detection software (Figure S2). The failure of improvement in mobility may be due to lack of effect in the mouse forelimb strength. An alternative hypothesis is that even though muscle strength is increased in the weak (hind) limbs, the animal endurance for sustained effort was not improved. In human studies muscle strength measurements (manual and quantitative) and mobility parameters (6 minute walk test) are separate outcome measures. In any case the lack of improvement in mobility even though muscle strength was improved and fibrosis was ameliorated may suggest that studying FTS as a component of a combination therapy is worthwhile. Future research direction may include studying the effect of FTS with corticosteroids, or alternatively with Glatiramer acetate which demonstrated improvement of strength and mobility in the same mouse model [24].

The mechanism of the beneficial effect of FTS in this model of muscular dystrophy is probably by decreasing inflammation and fibrosis as has been shown in experimentally induced liver cirrhosis, SLE, neuritis or nephritis animal models [13], [14], [15], [22], [23]. FTS has specificity towards Ras and Ras driven inflammatory and fibroblasts cell growth, with the advantage that it does not confer a global growth inhibitory effect on every type of cell [35]. Our results indicate that MMP-2 and MMP-9 collagenolytic activities are significantly increased in the untreated *dy^2J^/dy^2J^* compared to the WT groups and that FTS treatment resulted in significant inhibition of both MMP-2 and MMP-9 activities. These findings support previous data implying metalloproteinase activity is regulated by Ras activity and suggest that MMP- 2 & 9 inhibition is an important mechanism of fibrosis inhibition by FTS in the *dy^2J^/dy^2J^* mouse. Matrix metalloproteases (MMPs) play an important role during skeletal muscle degeneration, regeneration and in homeostasis and maintenance of myofiber functional integrity [36]. MMPs influence cell motility, interactions between cells, matrix degradation, tissue remodeling and the release of bioactive signaling molecules [37]. They are commonly induced by cytokines and secreted by inflammatory cells [38]. Over-expression of MMP-2 and MMP-9 on non-necrotic fibers may facilitate lymphocyte adhesion and enhance T-cell–mediated cytotoxicity by degrading ECM proteins [36]. MMP-2 plays essential roles in myofiber proliferation, differentiation, fiber healing after injury, and maintenance of the surrounding connective tissue [36], [39]. It is expressed in myoblasts and fibroblasts, within normal muscle tissue, and is over-expressed in the ECM in many pathological conditions in response to various initiating factors such as those occurring during inflammation, myopathies, muscle atrophy, and excessive exercise [36]. MMP-2 activity was found to be associated with the extent of pathological injury in *mdx* muscles, especially in the diaphragm [40] and increased MMP-2 activity was demonstrated in a patient with congenital muscular dystrophy [41]. MMP-9 up-regulation appears to be an important molecular event in the multi-step processes of muscle inflammation. It is strongly expressed in atrophic myofibers, activated satellite cells and in invading T lymphocytes [36], [38], [42], [43].

Li H. et al recently reported that the expression of MMP-9 is increased in dystrophic muscle and that its inhibition considerably reduced inflammatory response, fibrosis, and enhanced muscle regeneration in *mdx* mice [44]. Interestingly, our results differ from previous data of the liver cirrhosis rat model which showed increased MMP-2 and MMP-9 activities following FTS treatment. The discrepancy between the findings in different animals and diseases may also represent the fact that the role of each of these MMPs has been found to be divergent in different stages of disease progression [45].

Congenital muscular dystrophy with merosin deficiency is associated with central and peripheral nervous system dysmyelination. Both children and *dy^2J^/dy^2J^* mice have peripheral neuropathy in addition to progressive muscular dystrophy [7], [8]. Increased Ras activation was detected during Theiler's murine encephalomyelitis virus infection, a demyelinating disease that resembles human multiple sclerosis [46] and inhibition of Ras activation was suggested as a possible therapeutic directed against cytokine and NO mediated neuroinflammatory and neurodegenerative disorders [47]. An additional effect of Ras inhibition on the *dy^2J^/dy^2J^* peripheral neuropathy can therefore be postulated. This hypothesis was not evaluated in the current study.

The results of this study support previous findings that anti-inflammatory and anti-fibrotic agents have significant beneficial effect in the *dy^2J^/dy^2J^* and the *mdx* mouse models of muscular dystrophy. Treatment with Glatiramer Acetate, an immune modulating agent, resulted in significant improvement in hind limb muscle strength, improvement of mouse mobility as well as attenuation of the fibrosis markers vimentin and fibronectinin in *dy^2J^/dy^2J^* mice [25]. Halofuginone an anti-fibrotic agent (pro-collagen 1 inhibitor) treatment was associated with fibrosis inhibition in both *mdx* and *dy^2J^/dy^2J^* mice [48]. Losartan treatment resulted in improved muscle regeneration and diminished fibrosis in the *mdx* mouse by TGF-β antagonism. After six months treatment these mice showed increased strength and less muscle fatigue compared to sham treated mice [49].

The positive results of the current trial support further studying of FTS, an anti-fibrotic agent and a potent Ras inhibitor, as a potential new treatment in congenital and possibly additional forms of muscular dystrophy. Clinical studies using FTS for cancer are currently performed. Phase I/II study dosing FTS (salirasib)+gemcitabine in pancreatic cancer patients and a phase II trial of FTS treatment in lung cancer are underway (http://www.concordiapharma.com). Future studies in other animal models of muscular dystrophy such as the *mdx* mouse, FTS effect in combination with other medications and safety data in children are required towards consideration of studying FTS in human clinical trials.

## Supporting Information

Figure S1 **Fore limb muscle strength in FTS treated and untreated WT and** ***dy^2J^/dy^2J^*** **mice.** The data of seven mice in each group is expressed as mean ± SEM. Repeated measures ANOVA test showed no significant difference in the fore limb strengths between the treated and untreated *dy^2J^/dy^2J^* and WT groups. There was a significant difference between the WT and *dy^2J^/dy^2J^* fore limb muscle strength (*P*<0.01).
(TIF)

Figure S2 **The effect of FTS on mouse mobility.** The data of seven mice in each group is expressed as mean ± SEM. No significant difference was found between the treated and untreated *dy^2J^/dy^2J^* mice in maximal distance (A), maximal velocity (B) total distance (C) and mean velocity (D). Student's t-test and non parametric Mann-Whitney test showed significant difference in all 4 parameters between the WT and *dy^2J^/dy^2J^* untreated groups (*P*<0.001).
(TIF)
