# Supplementary figures and images for: The Ras Antagonist, Farnesylthiosalicylic Acid (FTS), Decreases Fibrosis and Improves Muscle Strength in dy2J/dy2J Mouse Model of Muscular Dystrophy
Source: PLoS One. 2011 Mar 22;6(3):e18049. doi: 10.1371/journal.pone.0018049 (PMC3062565; doi:10.1371/journal.pone.0018049)

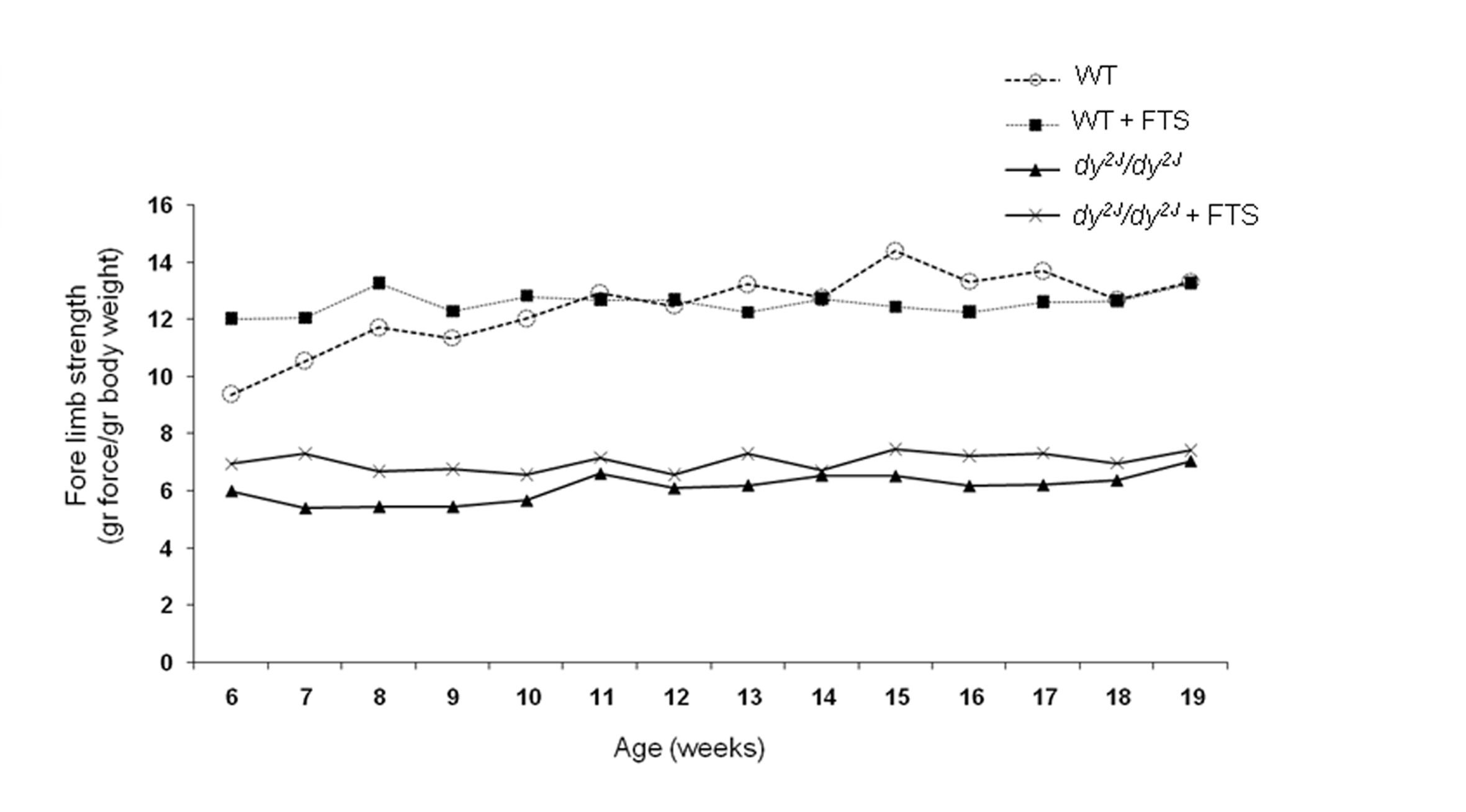

Supplement: Figure S1 — Fore limb muscle strength in FTS treated and untreated WT and dy2J/dy2J mice. The data of seven mice in each group is expressed as mean ± SEM. Repeated measures ANOVA test showed no significant difference in the fore limb strengths between the treated and untreated dy2J/dy2J and WT groups. There was a significant difference between the WT and dy2J/dy2J fore limb muscle strength (P<0.01). (TIF) [file pone.0018049.s001.tif]

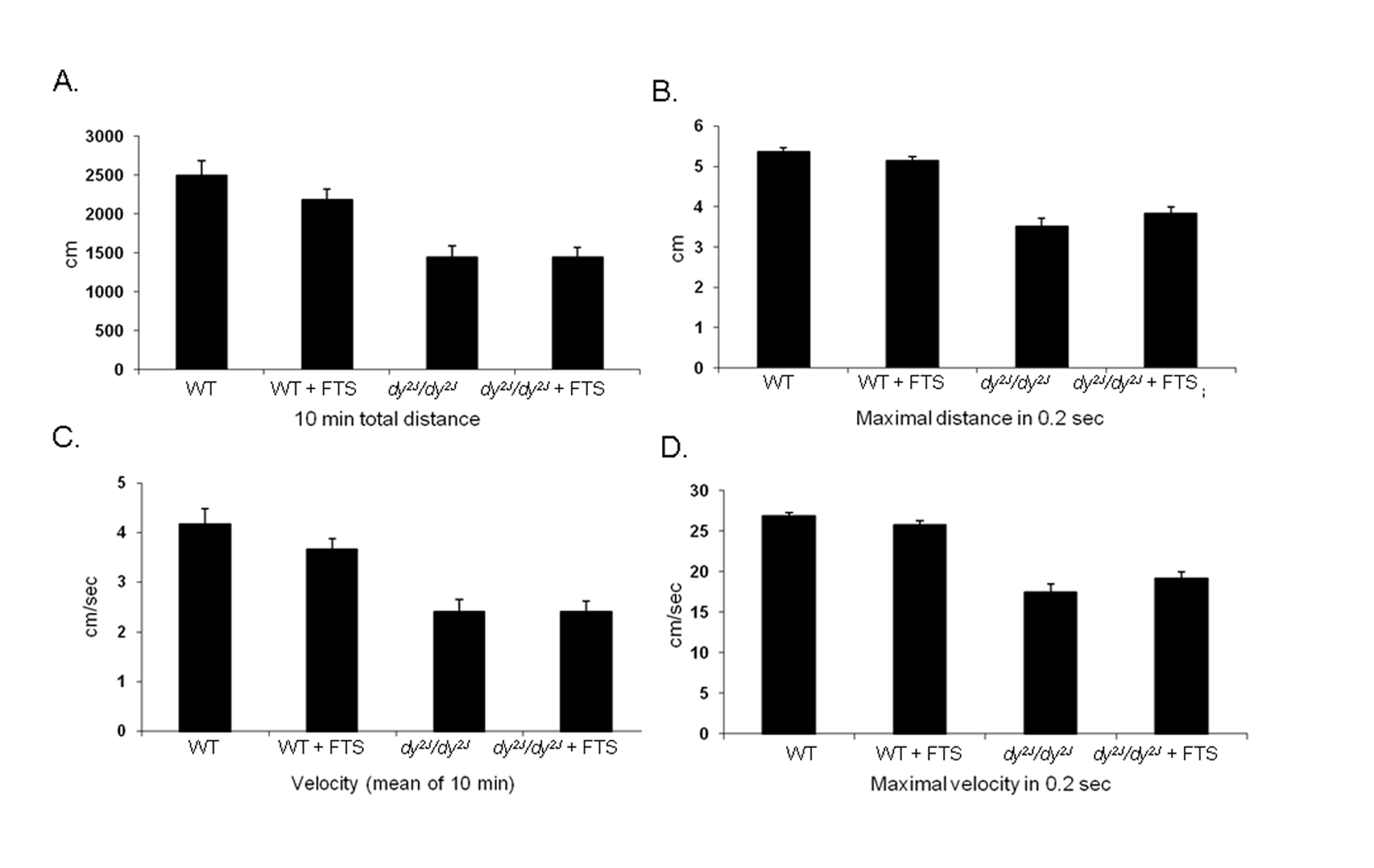

Supplement: Figure S2 — The effect of FTS on mouse mobility. The data of seven mice in each group is expressed as mean ± SEM. No significant difference was found between the treated and untreated dy2J/dy2J mice in maximal distance (A), maximal velocity (B) total distance (C) and mean velocity (D). Student's t-test and non parametric Mann-Whitney test showed significant difference in all 4 parameters between the WT and dy2J/dy2J untreated groups (P<0.001). (TIF) [file pone.0018049.s002.tif]
